# Antimicrobial Efficacy and Dentin Collagen Damage Caused by Calcium Hypochlorite and Sodium Hypochlorite

**DOI:** 10.1590/0103-6440202405771

**Published:** 2024-06-24

**Authors:** Ediléia Lodi, Yuri Dal Bello, Karen Barea de Paula Duarte, Francisco Montagner, Doglas Cecchin

**Affiliations:** 1 Universidade de Passo Fundo, Passo Fundo, RS, Brasil; 2 Universidade Federal do Rio Grande do Sul, Porto Alegre, RS, Brasil

**Keywords:** Confocal microscopy, calcium hypochlorite, dentin collagen, disinfection, polarized light microscopy, sodium hypochlorite

## Abstract

This study aimed to evaluate the antimicrobial activity of calcium hypochlorite (Ca (OCl)_2_) and sodium hypochlorite (NaOCl) using confocal laser scanning microscopy (CLSM) and dentin organic matrix alteration by picrosirius staining and light microscopy (LM). Samples of human extracted teeth were infected with *Enterococcus faecalis* by centrifugation of the bacterial suspension and were treated with Ca(OCl)_2_ or NaOCl at 0.5%, 2.5%, and 6% for 15, 30, and 60 seconds. CLSM and viability staining were used to quantitatively analyze the proportions of dead/live bacteria in the canal lumen and border of the root canal. The data were analyzed by ANOVA and Fisher test. For LM analysis, one hundred bovine teeth were randomly divided into 10 test groups (n=10): G1- Without treatment; G2- 17% EDTA; G3- 6% NaOCl; G4- 6% NaOCl + EDTA; G5- 0.5% Ca(OCl)_2_; G6- 0.5% Ca(OCl)_2_ + EDTA; G7- 2.5% Ca(OCl)_2_; G8- 2.5% Ca(OCl)_2_ + EDTA; G9- 6% Ca(OCl)_2_; G10- 6% Ca(OCl)_2_ + EDTA. The samples were fragmented and stained with Picrosirius. Data were analyzed by Kruskal-Wallis and Dunn (P<0.05). There was a strong correlation between the results of the canal lumen and the border of the root canal (r=0.962). Both hypochlorites at a concentration of 0.5% showed less microbial reduction compared to 2.5% and 6% (P<0.05). There was less antimicrobial activity at 15 seconds compared to 30 and 60 seconds (P<0.05). Ca(OCl)_2_ and NaOCl showed similar results at the same concentrations (P>0.05). In conclusion, Ca(OCl)_2_ caused fewer alterations to the dentin organic matrix at concentrations of 0.5% and 2.5%. Ca(OCl)_2_ presents antimicrobial activity similar to NaOCl, and collagen damage is concentration-dependent.

## Resumo

Este estudo teve como objetivo avaliar a atividade antimicrobiana do hipoclorito de cálcio (Ca(OCl)_2_) e do hipoclorito de sódio (NaOCl) usando a microscopia confocal a laser (MCVL) e a alteração da matriz orgânica da dentina por meio da coloração com picrosirius e microscopia óptica (MO). Amostras de dentes humanos extraídos foram infectados com *Enterococcus faecalis* por centrifugação da suspensão bacteriana. As amostras foram tratadas com Ca(OCl)_2_ ou NaOCl a 0,5%, 2,5% e 6% durante 15, 30 e 60 segundos. A MCVLe a coloração de viabilidade foram utilizadas para analisar quantitativamente as proporções de bactérias vivas/mortas no lúmen do canal e no bordo do canal radicular. Os dados foram analisados por ANOVA e teste de Fisher LSD. Para análise de MO, cem dentes bovinos foram divididos aleatoriamente em 10 grupos teste (n=10): G1- Sem tratamento; G2- 17% de EDTA; G3- 6% de NaOCl; G4- 6% de NaOCl + EDTA; G5- 0,5% de Ca(OCl)_2_; G6- 0,5% de Ca(OCl)_2_ + EDTA; G7- 2,5% de Ca(OCl)_2_; G8- 2,5% de Ca(OCl)_2_ + EDTA; G9- 6% de Ca(OCl)_2_; G10- 6% de Ca(OCl)_2_ + EDTA. As amostras foram fragmentadas e coradas com Picrosirius. Os dados foram analisados por Kruskal-Wallis e teste de Dunn (P<0,05). Houve uma forte correlação nos resultados do lúmen do canal e na borda do canal radicular (r=0,962). Ambos os hipocloritos, a uma concentração de 0,5%, mostraram menor redução microbiana em comparação com 2,5% e 6% (P<0,05). Houve menor atividade antimicrobiana aos 15 segundos em comparação com 30 e 60 segundos (P<0,05). Em conclusão, o Ca(OCl)_2_ e o NaOCl apresentaram resultados semelhantes nas mesmas concentrações (P>0,05). O Ca(OCl)_2_ causou menos alterações na matriz orgânica da dentina em concentrações de 0,5% e 2,5%. O Ca(OCl)_2_ apresenta atividade antimicrobiana semelhante ao NaOCl, e os danos ao colágeno dependem da concentraçã

## Introduction

Endodontic auxiliary chemical substances (ACS) used for preparing root canals should ideally present a broad antimicrobial spectrum and water solubility, dissolve necrotic remnants, have low toxicity for apical extrusion, and prevent smear layer formation during instrumentation or dissolve it after formation ^1^

Sodium hypochlorite (NaOCl) is the ACS most used for preparing root canals, as it effectively eliminates organic remnants and microorganisms organized in biofilms. This capacity is directly proportional to concentrations and exposure time ^2^. However, the higher the concentration, the greater the changes in mechanical properties, damage to dentin organic components ^3^ toxicity ^1^ ^,^ ^2^

Calcium hypochlorite (Ca(OCl)_2_) is a relatively stable chemical used in water purification and industrial sterilization ^4^. It has more active chlorine available than NaOCl ^5^, demonstrating antimicrobial efficacy and the ability to reduce *Enterococcus faecalis* in root canals ^6^ ^,^ ^7^. Furthermore, it seems less harmful to dentin mechanical properties ^8^ and exhibits a lower inflammatory response than NaOCl ^9^.

However, despite promising results, the disinfection capacity inside dentin tubules and the potential changes to root canal walls caused by Ca(OCl)_2_ have not been elucidated. Thus, this study analyzed and compared the efficacy of Ca(OCl)_2_ and NaOCl against *Enterococcus faecalis* infection and their effects on dentinal collagen fibrils. The tested hypothesis was that NaOCl and Ca(OCl)_2_ have similar antimicrobial capacity at the same concentrations. Regarding the impact on the dentin organic matrix, the hypothesis was that Ca(OCl)_2_ is less harmful than NaOCl.

## Materials and methods

This study was approved by the Research Ethics Committee in Humans (protocol no. 2.033.027) and the Ethics Committee on the Use of Animals for bovine teeth (protocol no. 005/2017).

### Confocal Laser Scanning Microscopy (CLSM)

Two hundred and fifteen human single-rooted teeth were selected. Teeth with complete root development, minimum root length of 14 mm, without dilacerations, severe curvatures, calcifications, and anomalies were used. They were kept frozen at -20°C until the research was conducted to preserve their properties. Five teeth were used for a pilot study to confirm and verify the viability of bacteria inside dentinal tubules, and 70 were used for the study.

### Specimen Preparation

The sample preparation followed the method adapted from Ma et al. ^10^ and Böttcher et al. ^11^ Crowns were removed using a double-sided diamond disc (S.S. White; Rio de Janeiro, RJ, Brazil) A root dentin block from the cervical portion with 4 mm in length, was horizontally sectioned from each tooth 1 mm below the cementoenamel junction using a diamond saw (Isomet 2000; Buehler Ltd, Lake Bluff, IL) at 800 rpm under water refrigeration. The samples were enlarged to a standardized canal diameter of 1.5 mm (±0.1 mm) using #6 Gates-Glidden drills (Dentsply Tulsa, Tulsa, OK) with a low-speed handpiece. Orientation grooves were made with diamond discs and each root was sectioned into 2 halves with a microtome blade (2 specimens per root, N = 420). Standardized dentin blocks (4 x 4 x 2 mm) were obtained. The inner and outer surfaces of the hemi-cylinders were flattened using 800-grain sandpaper under refrigeration (3M Brazil, Sumare, Brazil).

The samples were immersed in 5.25% NaOCl and then in 6% citric acid (pH 4.0) to remove the smear layer and rinsed with sterile distilled water for 1 minute with agitation and fixed with Putty-C Silicone for Impression (Silon2APS - Dentsply, Petropolis, RJ, Brazil) in plastic microtubes (Axygen Inc, Union City, CA, USA) to ensure they remained upright with the cervical portion facing upwards. Sterilization was performed at 120°C and 1 atm in an autoclave (Dabi Atlante, Ribeirão Preto, SP, Brazil) for 15 minutes.

### Culture and Inoculum Preparation

The reference strain used was *Enterococcus faecalis* (ATCC 29212). The bacteria were cultured in brain-heart infusion (BHI) broth for 18 to 24 hours at 37°C in a bacteriological incubator. The turbidity degree of the inoculum was adjusted using a spectrophotometer according to the MacFarland scale 1 (3 x 10^6^ CFU/ml) and an optical density of 0.25 at 510 nm 500µl of BHI broth suspension containing *Enterococcus faecalis* (3 x 106 CFU/ml) was inoculated into the root canal of each sample. The tubes were centrifuged at 1400g, 2000g, 3600g, and 5600g at low speed twice in each sequence for 5 minutes. After each centrifugation, the bacterial suspension was aspirated, discarded, and renewed with the addition of 500µl carried out in a laminar flow chamber. The dentin blocks were removed from the microtubes, and the surrounding residues from the dentin blocks were removed. All samples were incubated at 37°C in BHI broth for 24 hours after centrifugation to facilitate bacterial recovery.

### Classification of Treatment Groups

The samples were randomly divided into 42 experimental groups (n=10); (7 test substances x 3 times): 21 for evaluation in the canal lumen and 21 for the border of the root canal dentin. The substances and concentrations tested were: sodium chloride (NaCl) (control), NaOCl, and Ca (OCl)_2_ at concentrations of 0.5%, 2.5%, and 6% at contact times of 15, 30, and 60 seconds. A 50µl drop of each test substance was applied on the dentin surface at three different times. The samples were rinsed with sterile saline solution for 1 minute. The samples for root canal border dentin evaluation were fractured vertically into two halves using a microtome blade, exposing a new surface of the root canal dentin. One-half was used for evaluation.

### CLSM Analysis

The samples were individually placed on glass slides and stained with 10 μL of the Live/Dead BacLight Bacterial Viability Kit L13152 solution (Molecular Probes, Inc., Eugene, OR, USA), which was applied to the specimen according to the manufacturer's recommendations at a 1:1 ratio. The stained samples were examined using a confocal laser scanning microscope (CLSM Olympus Europa Holding GmbH, Hamburg, Germany), equipped with argon and helium-neon lasers, using a 60x objective lens. The excitation/emission wavelengths were 480/500 nm for SYTO 9 and 490/635 nm for propidium iodide. Images were obtained using the Olympus FluorView Version 4.2b software (Olympus Europa Holding GmbH, Hamburg, Germany) at a resolution of 512 x 512 pixels.

Fluorescence images captured from both the specimen section and the entire specimen were taken sequentially with the same gamma, brightness, and contrast settings for all samples. The evaluation of viable and non-viable cells was performed using the bioImageL v.2.1 software - Image Analysis Software for Investigation of Microbial Biofilms (The MathWorks, Inc., Natick, MA) following the protocol of Chávez de Paz ^12^, with images in TIFF (Tagged Image File Format) format. To discriminate microorganisms, the images were treated with contrast to highlight them. The images were separated by their green constituent (SYTO-9, viable cells) and red constituent (propidium iodide, non-viable cells). Two evaluations were made on two types of root dentin surfaces: the surface of the root canal lumen and the border of root canal dentin.

### Statistical Analysis (CSLM)

Data normality was assessed by the Kolmogorov-Smirnov test. The data were analyzed using a two-factor ANOVA test followed by the Fisher LSD multiple comparisons test. All data were analyzed at a significance level of 5% (p<0.05).

### Light Microscopy (LM)

One hundred bovine incisor teeth were used. Teeth were frozen at -20°C until the study. The root surface was sealed with two layers of cyanoacrylate (Super Bonder, Loctite, SP, Brazil). Crowns were cut 3 mm below the amelo-cemental junction using a double-sided diamond disc under water refrigeration. The canals were prepared with #5 and #6 Gates-Glidden drills and K #130 files (Maillefer, Ballaigues, VD, Switzerland) irrigated with sterile saline solution. The roots were randomly distributed into ten (n=10) experimental groups: Without treatment (Control); 17% EDTA; 6% NaOCl; Ca (OCl)_2_ 0.5%, 2.5%, and 6% associated or not with EDTA.

Irrigating solutions were applied using a 10-mL disposable plastic syringe (BD-Becton Dickinson, São Paulo, SP, Brazil) and a 30-gauge Endo-Eze needle (Ultradent Products Inc, South Jordan, UT). Needle penetration reached up to 3 mm short of the working length to allow irrigating solution reflux. The solutions remained in the root canal for 30 minutes, renewed every 3 minutes. In the combined groups, NaOCl or Ca (OCl)_2_ aspired to the root canal before introducing EDTA to avoid the direct mixing of solutions. All canals were dried with #80 absorbent paper points (Tanari; Tanariman Industrial Ltda, Manacapuru, AM, Brazil).

### LM analysis

After decalcification, dehydration, and inclusion in paraffin, 5-µm-thick slices were obtained and stained with picrosirius red. A total of 30 slides were stained for each group. Each slice was examined under a fluorescence polarization microscope, Axio Vert A1 FL-LED (Zeiss Microsystems GmbH, Wetzlar, Hesse, Germany), where the most representative area of birefringence pattern was captured at magnifications of 40x, 100x, 200x, and 400x by a calibrated operator (Kappa = 0.873) blinded to the experimental groups.

The evaluation of collagen damage, performed on the microscopy images, followed the method used by Ghisi et al. 2015 ^3^ according to the following scores:

Score 0: Organized collagen fibers, arranged parallel to each other, and homogeneous contour of the root canal lumen.

Score 1: Collagen fibers showing an altered discontinuous pattern and irregular contour of the root canal lumen in up to 25% of the analyzed area.

Score 2: Collagen fibers showing an altered discontinuous pattern and irregular contour of the root canal lumen in up to 50% of the analyzed area.

Score 3: Collagen fibers showing an altered discontinuous pattern and an irregular contour of the root canal lumen in more than 50% of the analyzed area.

Score 4: Collagen fibers showing an altered discontinuous pattern and irregular contour of the root canal lumen throughout the entire analyzed area.

### Statistical Analysis (LM)

The final score for each sample corresponded to the root canal slice in which the damage to the organic compound was most pronounced. Statistical analysis was performed using SigmaPlot 14.0 software (Systat Software Inc., USA). The Kruskal-Wallis test and post hoc Dunn test were applied at a significance level of 5%.

## Results

### CSLM

**Figure 1:**
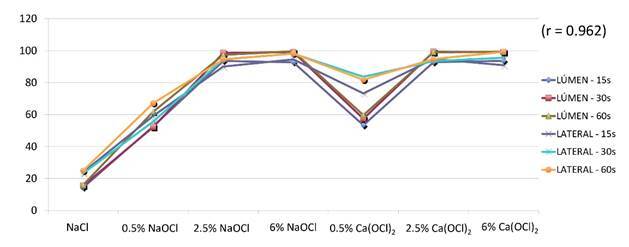
Correlation of data evaluated in the lumen of the canal and lateral dentin surface.

Statistical analysis of the data showed a statistically significant difference between groups at both evaluation sites (canal lumen and border of root canal) (p < 0.027). Additionally, a strong correlation was observed between the evaluation sites (r = 0.962) (Figure 1).

The data from Tables 1 and 2 demonstrate the decontamination capacity of different solutions at different times in the root canal lumen and border of a root canal, respectively. In the root canal lumen, it is possible to observe that decontamination was superior at concentrations of 2.5% and 6% (p < 0.05) for both hypochlorites compared to the control and the 0.5% concentration, regardless of the contact time. The best action of NaOCl at a concentration of 0.5% was observed at 60 seconds (p < 0.05). Except for the control group and the 0.5% sodium hypochlorite, all substances improved their antimicrobial action at 30 and 60 seconds (p < 0.05). Similar results were observed when the evaluation was performed on the root border of the canal (Table 3), where concentrations of 2.5% and 6% showed statistically higher antimicrobial effects (p < 0.05). In the control group, the lowest percentage of dead cells was observed among all groups (p < 0.05) at both evaluation sites.

**Table 1:** Percentage of dead cells in dentinal tubules with different irrigating solutions - media and standard deviation values in the root canal lumen

Solution	Concentration (%)	Time (sec.)		
		15 s	30 s	60 s
NaCl	0.9	14,38±2,12Bc	15,53±1,74Bc	15,97±0,99Bc
NaOCl	0,5	52,61±13,63Bb	52,18±4,19Bb	61,81±3,31Ab
	2,5	93,58±5,02Ba	98,59±1,50Aa	97,34±2,43Aa
	6	92,74±3,56Ba	99,13±1,06Aa	99,63±0,65Aa
Ca (OCl)_2_	0,5	53,34±8,23Bb	57,39±6,91Ab	59,65±7,55Ab
	2,5	92,82±6,33Ba	99,29±0,95Aa	98,98±1,35Aa
	6	93,60±5,11Ba	99,07±1,41Aa	99,27±0,81Aa

*NaCl, sodium chloride; NaOCl, sodium hypochlorite; Ca(OCl)2, calcium hypochlorite.*

*Medians followed by different uppercase letters in the same row are statistically different (p < 0.05). Medians followed by different lowercase letters in the same column are statistically different (p < 0.05).

**Table 2:** Percentage of dead cells in the dentinal tubules with different irrigating solutions media values and standard deviation observed in the border of root dentin.

Solution	Concentration (%)	Time (sec.)		
		15 s	30 s	60 s
NaCl	0.9	14,38±2,12Bc	15,53±1,74Bc	15,97±0,99Bc
NaOCl	0,5	52,61±13,63Bb	52,18±4,19Bb	61,81±3,31Ab
	2,5	93,58±5,02Ba	98,59±1,50Aa	97,34±2,43Aa
	6	92,74±3,56Ba	99,13±1,06Aa	99,63±0,65Aa
Ca (OCl)_2_	0,5	53,34±8,23Bb	57,39±6,91Ab	59,65±7,55Ab
	2,5	92,82±6,33Ba	99,29±0,95Aa	98,98±1,35Aa
	6	93,60±5,11Ba	99,07±1,41Aa	99,27±0,81Aa

*NaCl, sodium chloride; NaOCl, sodium hypochlorite; Ca(OCl)2, calcium hypochlorite.*

*Medians followed by different uppercase letters in the same row are statistically different (p < 0.05). Medians followed by different lowercase letters in the same column are statistically different (p < 0.05).

**Table 3:** Scores and medians of the changes in dentin collagen caused by the test substances

Groups	Score 0	Score 1	Score 2	Score 3	Score 4	TOTAL	Median (Mín-Máx)*
Control (without treatment.)	48	1	0	0	0	49	0,0 (0-3) a
17% EDTA	31	4	0	0	0	35	0,0 (0-3) a
6% NaOCl	1	3	4	12	24	44	3,5 (0-4) d
6% NaOCl + EDTA	2	5	6	5	23	41	4,0 (0-4) d
0,5 % Ca(OCl)_2_	16	16	1	1	0	34	1,0 (0-3) ab
0,5% Ca(OCl)_2_ + EDTA	33	20	4	4	0	61	0,0 (0-3) a
2,5 % Ca(OCl)_2_	35	11	2	3	3	54	0,0 (0-4) a
2, 5% Ca(OCl)_2_+ EDTA	30	9	3	4	3	49	0,0 (0-4) a
6% Ca(OCl)_2_	7	14	8	10	8	47	2,0 (0-4) cd
6% Ca(OCl)_2_ + EDTA	10	16	15	5	5	51	1,5 (0-4) cb
Total	213	99	43	44	66	465	

*The data represents the number of evaluations (images evaluated) in each group in terms of the scores observed in dentin collagen (total per group and per score evaluated) and the total of all evaluations.*

*Medians followed by equal letters are statistically similar (p≥0.05).

### LM

The data were evaluated according to the collagen damage pattern found around the root canal. There was a significant difference between the groups in terms of collagen alteration degree (P < 0.0001). The results show that the damage caused to dentin by Ca (OCl)_2_ at concentrations of 0.5% and 2.5% (Table 3) was statistically similar to EDTA and the control group (p > 0.05). At 6% concentration, Ca (OCl)_2_ was statistically similar to 6% NaOCl, and when associated with EDTA, it was observed less damage to collagen fibers than 6% NaOCl (p < 0.05). Thus, confirming partially the study's hypothesis regarding dentin's organic matrix damage. EDTA only showed statistically similar results to the control group with scores between 0 and 1. It is possible to observe that the damage caused by Ca(OCl)_2_ increased with increasing concentration, and the association with EDTA did not increase the damage.

## Discussion

This study suggested that lower concentrations of the tested substances took longer to achieve high dentin disinfection. These findings agree with other studies in the literature ^16^ ^,^ ^17^ ^).^ Ma et al. ^10^ tested contact times of one and three minutes for 1%, 2%, and 6% NaOCl, showing that the proportion of dead bacteria inside dentinal tubules depended on the exposure time and concentration of the tested substances. It is worth noting that our study found that 2.5% and 6% concentrations for both hypochlorites (NaOCl and Ca(OCl)_2_) were statistically superior to 0.5% (p < 0.05). Furthermore, the antimicrobial capacity was similar when comparing substances at the same concentrations (Tables 2 and 3), confirming the first hypothesis. Similar findings appeared in a study where Ca(OCl)_2_ demonstrated equivalent antimicrobial activity to NaOCl at 2.5% and 5.25% concentrations ^7^. Besides this antimicrobial activity, Ca(OCl)_2_ dissolves pulp tissue, performing similarly to NaOCl ^15^.

Confocal laser scanning microscopy (CLSM) is an assessment method for the antimicrobial capacity of irrigating solutions that investigates and quantifies bacterial viability ^12^ ^,^ ^13^. Our study evaluated the effect of Ca(OCl)_2_ and NaOCl on dentin disinfection and collagen damage, analyzing these substances in direct contact with the root dentin. This methodology assesses bacterial depth and viability in dentinal tubules ^10^ ^,^ ^14^. Antimicrobial action was analyzed without associations with EDTA because the aim was to investigate the action of only the test substances. Both hypochlorites had similar antimicrobial activity at the same concentrations but did not eliminate *Enterococcus faecalis* from root dentin tubules. A study with a comparable methodology ^18^ found equivalent results.

CLSM may exhibit autofluorescence in organic debris in the canal lumen, which can be mistaken for bacteria. Therefore, Ma et al. ^10^ proposed evaluating antimicrobial activity with CLSM at the dentin border. In this methodology, samples require fracturing after treatment to expose a fresh dentin surface. The present study analyzed the canal lumen and root dentin border, finding a strong correlation between the evaluation sites (Figure 1). Tables 1 and 2 also show similar results for the tested substances at both sites, suggesting that the evaluated surface did not significantly influence the results.

The action mechanism of Ca(OCl)_2_ explains our findings, showing the following reaction: Ca(OCl)_2_ + 2H_2_O → 2 HOCl + Ca(OH)_2_ ^5^ ^,^ ^19^_._ During dissociation, Ca(OCl)_2_ releases two molecules of hypochlorous acid (HOCl), whereas NaOCl releases only one ^5^. Therefore, the chlorine concentration is higher in Ca(OCl)_2_, whose content is proportional to increases in concentration. The chlorine level is related to its disinfection capacity, and the literature has reported saponification and chloramination reactions for NaOCl ^2^. However, we did not find significant differences between the groups testing NaOCl and Ca(OCl)_2_ solutions at the same concentrations, corroborating previous studies ^6^ ^,^ ^7^. The higher surface tension of Ca(OCl)_2_ justifies our data, as this characteristic may affect substance penetration into dentinal tubules ^19^. The present study measured surface tension before light microscopy evaluation, showing 73.3 mN/m for 0.5% Ca(OCl)_2_, 74.2 mN/m for 2.5%, and 76.0 mN/m for 6%, and 66.7 mN/m for 6% NaOCl, 28.0 mN/m for EDTA, and 73.5 mN/m for NaCl (control).

The Picrosirius-polarization method measures the amount of collagen in normal or pathological tissues, revealing the molecular order, organization, and/or heterogeneity of collagen fibers in different tissues and determines developmental and repair processes in dental and bone tissues, representing an accurate, sensitive, and specific method for analyzing collagen. The parallel fiber orientation makes them visible under light microscopes because they exhibit birefringence ^20^. Picrosirius enhances birefringence, as collagen molecules are rich in basic amino acids that strongly react with the dye molecule, which is acid ^21^.

This study used bovine incisors because of the similar number of tubules in regular human and bovine dentin, considering they have the same collagen organic matrix and bovine dentin has a higher mineral composition homogeneity than human dentin ^22^ ^,^ ^23^. Furthermore, animal age is easily controlled, allowing for result standardization.

This study compared 0.5%, 2.5%, and 6% Ca (OCl)_2_ with 6% NaOCl. A previous study evaluated 2% and 5% NaOCl, finding collagen damage proportional to the increase in concentration ^3^. In the present study, 6% Ca (OCl)_2_ was statistically similar to 6% NaOCl, and when associated with EDTA, 6% Ca (OCl)_2_ caused lower damage to collagen fibers than 6% NaOCl (p < 0.05). Thus, the second hypothesis of the study was partially rejected.

Although NaOCl is extensively used for preparing root canals, it demonstrates an adverse effect on dentin collagen ^24^. NaOCl disorganizes structural collagen fibers potentially related to the tissue dissolution capacity promoted by the combined action of HOCl and NaOH on the dentin organic matrix ^25^. Thus, NaOCl releases HOCl (a weak acid) and NaOH (a strong base) in an aqueous medium ^2^. Chlorine is a strong oxidant with antimicrobial action, and when HOCl contacts the organic tissue, it shows a solvent action. HOCl- and hypochlorite ions (OCl-) cause amino acid degradation and hydrolysis. It is worth noting that such a relationship depends on the concentration, contact time, and volume of solutions ^2^. However, high concentrations and long exposure times may increase the deleterious effects on dentin components ^2^ ^,^ ^14^, potentially explaining the significant structural collagen disorganization in groups using NaOCl.

The association of 17% EDTA with both hypochlorites did not alter the degree of collagen degradation compared to the isolated use of these substances (Table 3), as EDTA changes the inorganic portion of dentin, corroborating previous studies ^3^ ^,^ ^24^. Furthermore, the control group and EDTA did not differ, confirming that EDTA does not affect the organic portion of dentin.

Due to NaOCl limitations, Ca (OCl)_2_ seems a promising alternative as an irrigating solution. This substance demonstrated lower cytotoxic effects ^9^, similar antimicrobial effectiveness to NaOCl ^6^ ^,^ ^7^, and collagen damage comparable to or even lower than NaOCl when combined with 17% EDTA. However, this is an *in vitro* study, and the results should be carefully interpreted. Randomized clinical trials are required to establish Ca (OCl)_2_ as a viable alternative irrigant in endodontic therapy.

Considering the limitations of this study, the CLSM evaluation site did not affect the results. NaOCl and Ca (OCl)_2_ at 2.5% and 6% showed high antimicrobial activity. Ca (OCl)_2_ presented a similar antimicrobial activity to NaOCl at the same concentrations, and collagen damage was concentration-dependent. The 2.5% Ca (OCl)_2_ showed high antimicrobial capacity with minimal collagen damage.
